# Visualizing Pathways to HIV Prevention for Black Women in Orange County, Florida: A Concept Mapping Study

**DOI:** 10.1007/s40615-025-02569-5

**Published:** 2025-10-10

**Authors:** J. Richelle Joe, Shan-Estelle Brown, Andrea Dunn

**Affiliations:** 1 Department of Counselor Education and School Psychology, University of Central Florida, 12494 University Boulevard, Orlando, FL 32817 USA; 2 Rollins College, Orlando, FL USA; 3 Let’s Beehive! Inc, Orlando, FL USA

**Keywords:** HIV prevention, PrEP, Black cisgender women, Group concept mapping

## Abstract

The National HIV/AIDS Strategy identifies Black cisgender women as a key population for HIV prevention, while the Ending the HIV Epidemic initiative names Orange County, Florida, as a priority jurisdiction. Although Black cisgender women are overrepresented among new HIV diagnoses, they are underrepresented among recipients of PrEP services. The simultaneous and overlapping racial and gender identities of Black cisgender women have historically placed them in a precarious position when seeking healthcare, including sexual health information and services. In an effort to dismantle this complex form of systemic racism, our interdisciplinary team conducted a community engaged research project using group concept mapping to visualize methods of facilitating comprehensive HIV prevention for Black cisgender women in Orange County, Florida with specific actionable steps for healthcare providers and community stakeholders. Community stakeholders attended nine stakeholder meetings, during which they generated, sorted, and rated 79 statements in response to the prompt *How can everyone in Central Florida improve Black women’s sexual health, create satisfying healthcare experiences for them, and enhance HIV prevention services?* Using multi-dimensional scaling and hierarchical cluster analyses, we generated a concept map with nine clusters which was discussed with community stakeholders to develop a plan for advancing HIV prevention among Black women in Orange County, Florida.

The National HIV/AIDS Strategy identifies Black cisgender women as a key population for HIV prevention [1]. The national prevalence rate for Black cisgender is disproportionately high [2], and this HIV disparity is associated with multiple social, structural, and geographic factors [3]. Within priority jurisdictions identified in Ending the HIV Epidemic in the US (EHE) initiative, incidence and prevalence of HIV for key populations is a particular concern, and Orange County, Florida (FL), is one such jurisdiction [4]. In this region, Black cisgender women rank third for new HIV diagnoses after Black and Latine individuals reporting male to male sexual contact [5]. This population is overrepresented among new HIV diagnoses and underrepresented among recipients of pre-exposure prophylaxis (PrEP) services [6].

The simultaneous and overlapping racial and gender identities of Black cisgender women have historically placed them in a precarious position when seeking healthcare, including sexual health information and services [7, 8]. The gendered racism that Black cisgender women face when navigating their sexual health needs is fueled by sexual stereotypes that depict Black women as oversexualized, asexualized, defeminized, and dehumanized [9–11]. In an effort to dismantle this complex form of systemic racism, our interdisciplinary team conducted a community engaged research project using group concept mapping to visualize methods of facilitating comprehensive HIV prevention for Black cisgender women in Orange County, FL with specific actionable steps for healthcare providers and community stakeholders.

The *Prevent* pillar of the EHE initiative focuses on methods to decrease HIV incidence across regions and populations via evidenced-based interventions, including PrEP [4]. Although PrEP has been approved by the US Food and Drug Administration since, 2012, its uptake has been uneven across demographic groups, with Black people and women being among the lowest users of the medication. Despite accounting for 39% for new HIV diagnoses, only 14% of PrEP users are Black. Moreover, only 8% of PrEP users are female [12, 13]. Hence, Black women account for a dismally low number of individuals on PrEP despite them accounting for more than half of new HIV diagnoses among women.

Although Black women report similar perceived risk of HIV as their white counterparts, they are more likely to test for HIV but less likely to be knowledgeable of PrEP [14]. In addition to lack of knowledge, low acceptability of PrEP among Black women is associated with stigma and the misconception of PrEP as a medication only for gay men [15]. Black women often report low perceived risk of HIV, which coupled with anticipated HIV or PrEP stigma held by partners and others in their social networks, can result in their lack of PrEP uptake [16, 17]. Even for Black women interested in PrEP, barriers to engagement with PrEP services include multiple structural and syndemic factors, such as the experience of community or interpersonal violence and poor access to necessary daily living services, like childcare and transportation [18]. Healthcare workers also cite inadequacies within healthcare systems (including lack of clinic infrastructure and insufficient human resources) and the cost of PrEP as barriers to their efforts to prescribe PrEP. [15]. Moreover, historical factors also shape Black women’s PrEP engagement. Among both Black and white women, medical mistrust is negatively associated with comfort discussing PrEP with a healthcare provider, and this mistrust is significantly higher among Black women [14]. Healthcare providers’ willingness to discuss and prescribe PrEP to Black women continues to be a barrier to PrEP uptake within this population and is associated with providers’ racial bias and perceptions of Black women’s potential adherence to the medication [19].

## Study Purpose

The EHE initiative focuses on HIV prevention, response, and care services in 57 priority jurisdictions that account for a substantial number of new HIV diagnoses [4]. Research exploring HIV prevention and PrEP initiatives for Black women are similarly localized and community-engaged [20–25]. Such studies provide insight into the geographical nuance of design and implementation of PrEP services for Black women. Although Orange County, FL is a priority jurisdiction along with Washington, D.C. [23], Chicago [21], and Mississippi [24], the sexual health experiences and needs of Black women in this region have been unexplored. As a result, HIV incidence remains high in this population while PrEP usage remains low. Therefore, we sought to employ a localized, community-driven research approach to address the unknown and ignored sexual health and HIV prevention concerns of Black cisgender women in Orange County, FL.

## Method

Our research team used group concept mapping (GCM) as a community-based participatory research (CBPR) method to engage community stakeholders around the topic of concern: Black cisgender women’s sexual health and HIV prevention needs. GCM was the second phase of a three-year parent project which had an overarching purpose of determining how local healthcare providers could prioritize the sexual health and HIV prevention needs of Black cisgender women in their services in alignment with the Orange County, Florida Ending the HIV Epidemic plan [26]. The first phase of our project included individual interviews with Black cisgender women living in the designated region and local healthcare providers, including individual practitioners as well as employees of non-profit HIV-serving organizations. The GCM phase of the project built upon these interviews in that the data were used to support the brainstorming processes in response to the focus prompt *How can everyone in Central Florida improve Black women’s sexual health, create satisfying healthcare experiences for them, and enhance HIV prevention services?* All activities for the GCM phase occurred under the protocol for the parent project, which was approved by a university institutional review board.

### Research Team

Known locally as Team Orlando, the research team included two researchers and one community partner, all of whom were equal partners in the project as required by the grantor. The community partner serves as the executive director of Let’s Beehive! Inc., a local non-profit organization that provides HIV education, prevention, and support services for youth and women. Let’s Beehive! was founded in 2015 and since that time, has hosted an annual HIV awareness symposium marking National Women and Girls HIV/AIDS Awareness Day, facilitated status-neutral women’s sexual health support groups, and led virtual stigma-reducing support groups for people living with HIV. The two researchers on Team Orlando represent the fields of counselor education and medical anthropology and have engaged in research that positions HIV as both a mental health and public health issue. All three members of the team identify as Black cisgender women which shapes their understanding of the healthcare and HIV-associated concerns facing the target population. All members of the team contributed to the determination of the research focus and approach, and all were involved in the GCM process.

### Group Concept Mapping

GCM is a mixed methods approach that first uses qualitative (group process, brainstorming, unstructured sorting, interpretation) followed by quantitative (multidimensional scaling, hierarchical cluster analysis) methods to graphically represent a topic of interest [27, 28]. As a CBPR tactic, GCM includes stakeholders in shared decision-making where they are invited to contribute ideas, systematically organize those ideas, and then represent those ideas in pictures and maps so that complex problems can be more easily understood [27, 29]. Through sustained involvement of stakeholders, GCM upends existing power hierarchies and promotes empowerment instead of data extraction from individuals and communities. A solid body of literature exists indicating the effective use of GCM to explore various public health concerns, including cervical cancer screening, chronic disease disparities, HPV vaccinations, and HIV prevention [30–34].

### Procedures

GCM is a six-step process that results in a visual representation of data that can be used to determine action steps or policy and program implementation that can address the central concern [27]. Our study followed these steps (preparation, generation, structuring, representation, interpretation, and utilization) with the central concern being Black cisgender women’s sexual health and HIV prevention needs. An exhaustive description of the steps of GCM is beyond the scope of this paper; however, additional details about the process can be found in the developer’s text on the subject [27].

### Recruitment and Participants

During the preparation step, we identified the project’s focus and established our logistical plan to include timeline, schedule, setting, and data management. Although Black cisgender women and their HIV prevention needs were identified as the focus area, we determined that the stakeholders to be involved in this study should be inclusive of other racial and gender identities. We made this determination because Black women’s sexual health is often connected to men’s health, as heterosexual contact is the primary mode by which Black women contract HIV. Additionally, given the current healthcare workforce, we recognized the need to open the study to providers, many of whom are neither Black nor women. Hence, our inclusion criteria included 1) being at least 18 years of age, 2) residing in the local community (to include Orlando and surrounding counties), and 3) invested in Black women’s health. We used non-probability and snowball sampling to recruit participants at local EHE meetings and community health events and through community organizations and interpersonal contact. Additionally, individuals who had completed an interview during the first phase of the larger study were also invited to participate in the GCM process. Thirty individuals expressed interest in being a stakeholder, and ultimately *N* = 19 stakeholders were involved in the meetings and completed the tasks necessary to create the concept maps. Although gender was not an inclusion criterion, all participants identified as women. Additionally, 84% (*N* = 16) identified as Black or African American, with 7 participants identifying as Caribbean, 3 specifically as Haitian, and 3 as African. This ethnic diversity was important given the corresponding diversity among Black women in the region. More than half of the participants (52.63%, *N* = 10) were in the age range of 25–34. Nearly 58% of participants (*N* = 11) reported being single (never married) and 52.63% (*N* = 10) reported no sexual activity at the time of the study.

### Stakeholder Meetings

Following the preparation step, the remaining five steps of the GCM process took place through a series of nine stakeholder meetings held at the Let’s Beehive! office, a private, easily accessible space known to members of the community. Attendance at the stakeholder meetings ranged from 8 to 16 participants (*m* = 12.8) depending on participants’ availability. Our first stakeholder meeting included an introduction to the team and the research project as well as an orientation to GCM as a research method. Recognizing that stakeholders would have varying levels of knowledge about Black women’s HIV vulnerability, we included surveillance data and basic HIV information to ensure a baseline of common knowledge in the group. During the second stakeholder meeting, we began the generation step of GCM, which included the discussion and finalization of prompts that would be used for brainstorming. Although brainstorming occurred in a group setting, each stakeholder was invited to contribute their own ideas by writing them out individually. To facilitate the brainstorming process, we drew from the interview data in phase one of the larger study to share de-identified quotes and fictional personas based on Black cisgender women in the region. Participants generated statements during this step, which the research team reviewed and edited for grammar, spelling, and redundancy. This resulted in a final set of 79 statements that we brought back to the stakeholders for review. The generation step spanned stakeholder meetings two through five, and in the fifth meeting we prepared the stakeholders for the structuring step. During this step, we also facilitated a discussion about how the statements should be rated to yield the most meaningful concept map. Eight dimensions were proposed: importance, changeability, feasibility, effectiveness, community support, impact, easiness/likeliness, and complexity. Of these eight, the participants selected importance, feasibility, and impact as the most meaningful scales.

Stakeholder meeting six was dedicated to stakeholders using The Concept System® groupwisdom™ software (Build 2022.30.10) to sort the 79 statements into meaningful groups and to rate them on a five-point Likert-type scale for the selected dimensions. Participants who were not able to attend this meeting in person were permitted to complete the task at home using their own device. All other participants used researcher-supplied devices in the Let’s Beehive! office. After this meeting, we used the groupwisdom™ software to analyze the data (representation step) and represent participant responses as graphs and clusters. Through multi-dimensional scaling and hierarchical cluster analyses, we determined the most parsimonious and meaningful organization of the statements into clusters. The groupwisdom™ software allowed us to configure the 79 statements in two-dimensional space with the underlying assumption that statements sorted similarly by stakeholders would be depicted closely in contrast to statements grouped together less often [28]. The remaining stakeholder sessions were used for the interpretation and utilization steps of GCM. The stakeholders collaborated with us to examine the output of the data analysis, identify the meanings of the clusters, name the clusters, and decide how to implement the information generated through the GCM process.

## Results

**Fig. 1:**
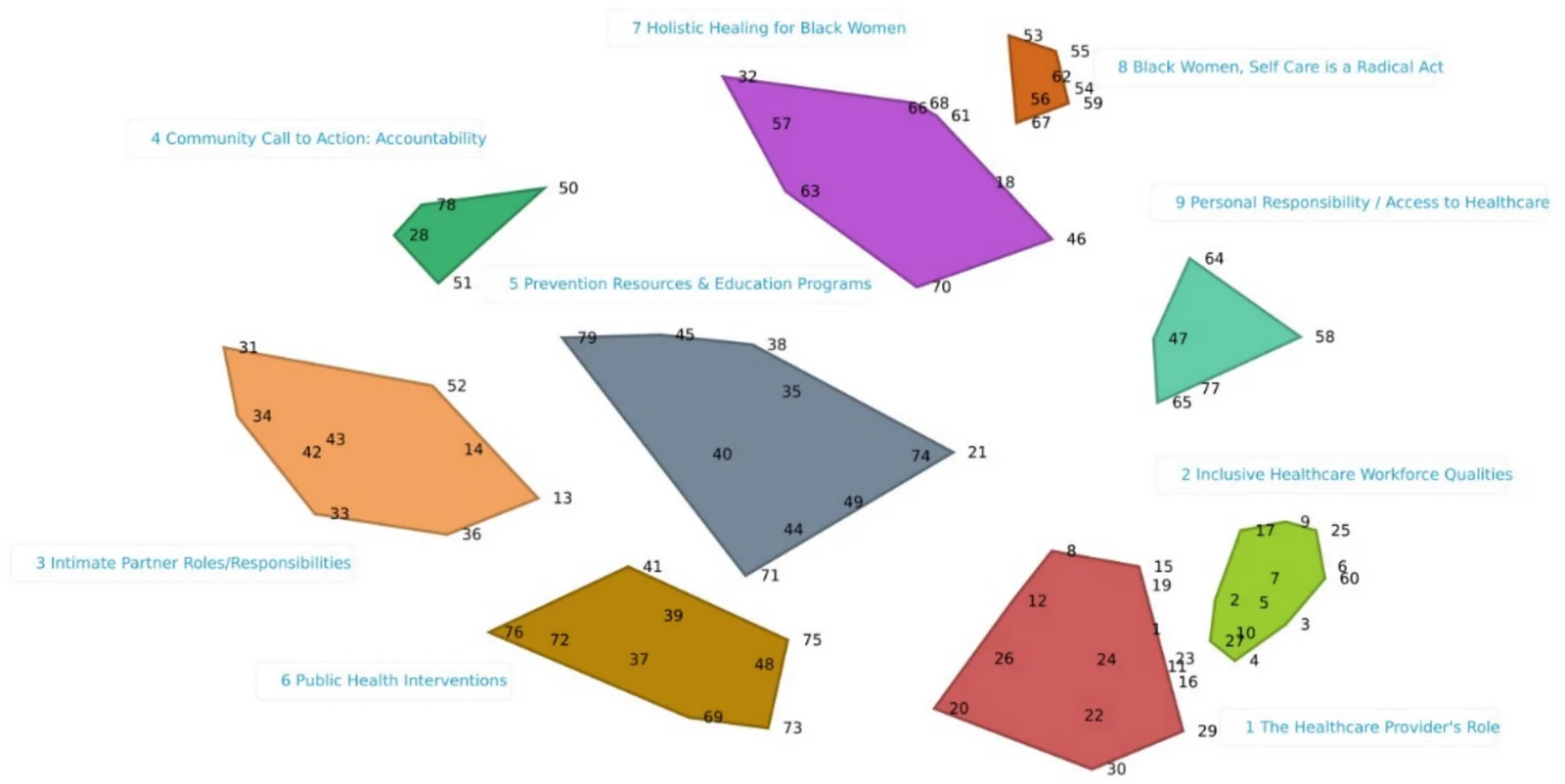
Cluster map of 79 statement point map

**Fig. 2:**
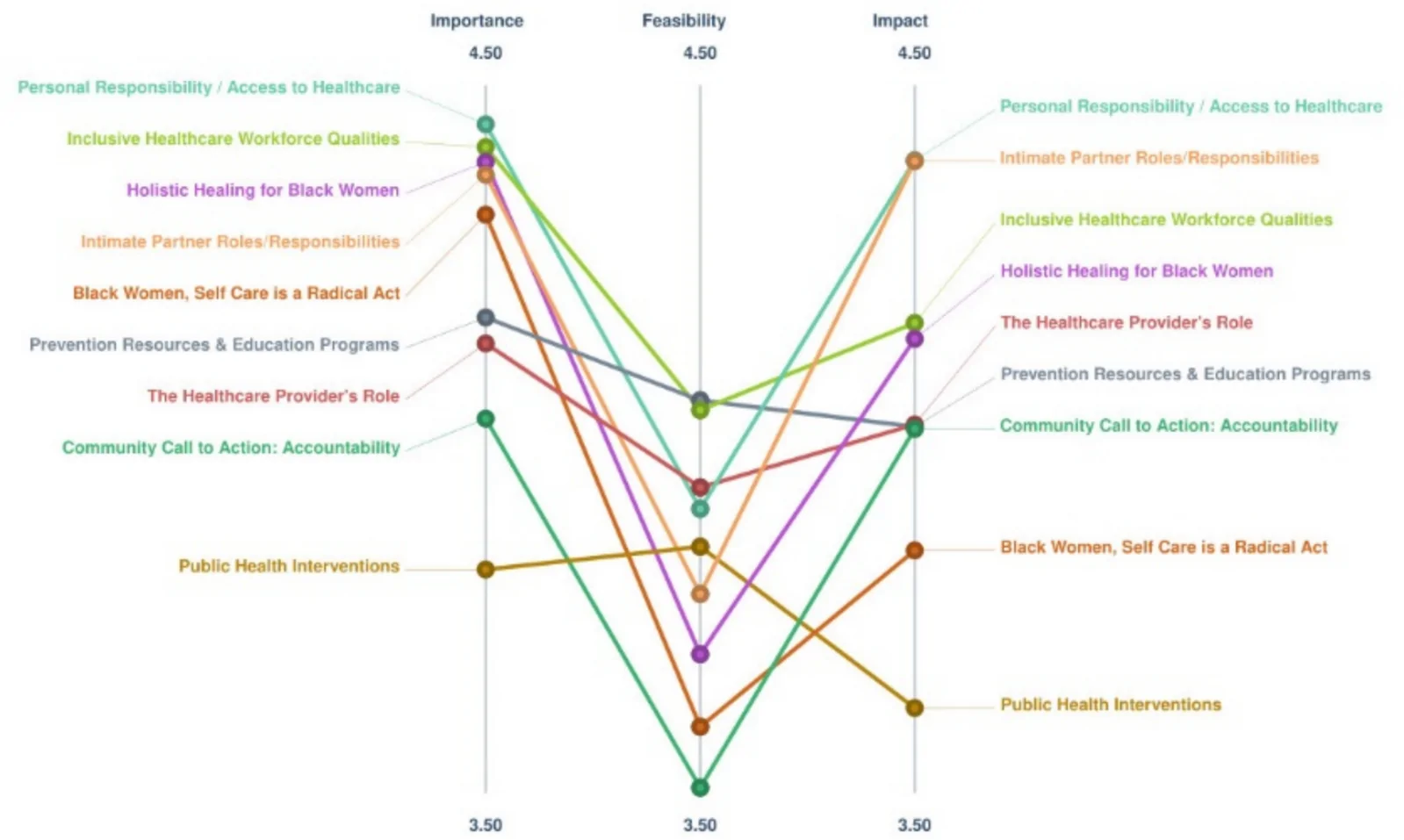
Pattern match graph

The GCM process yielded multiple visual representations of the data that were discussed with the stakeholders to determine their meaning and use. A point map depicted the relational structure of the brainstormed statements, and the cluster map (Fig. 1) provided organization of the statements into nine non-overlapping clusters. Although the shape of the clusters in GCM is arbitrary, the size of each cluster indicates the cohesiveness of the meaning represented by that cluster [28]. Our data analysis also resulted in a pattern match graph (Fig. 2) that demonstrated the relationships among the clusters based on the stakeholders’ ratings of importance, feasibility, and impact.

### Cluster Map

The nine clusters on the cluster map indicate proposed ideas regarding collective action to improve Black women’s sexual health, create satisfying healthcare experiences for them, and enhance HIV prevention services. In our discussions of these clusters, we determined that some clusters were relevant for healthcare providers while others were either directed toward Black women or included a shared community response to address HIV from a public health perspective. Additionally, whereas some individual statements were specific (e.g., Healthcare provider training on PrEP), others were vague, requiring them to be understood in relation to other statements (e.g., Inclusive marketing). Below is a discussion of the clusters and their corresponding statements.

### The Healthcare Provider’s Role in Black Women’s HIV Prevention

This cluster included statements directed toward healthcare providers serving Black women and addressed providers’ knowledge and behavior as well as logistical aspects of healthcare access. The provider’s role included the ability to recognize structural racism and how it shapes health outcomes for Black patients, with one strongly worded statement reading “unlearn racist stereotypes taught in medical school.” Discussion of that statement included mistaken beliefs about Black bodies and pain tolerance. Statements in this cluster also addressed the proximity of care providers to underserved communities, multiple lines of communication, and an overall environment that is welcoming to Black women. Statements specific to HIV prevention indicated a need for healthcare providers to be forthcoming about HIV testing, prevention, and treatment options.

### Qualities of an Inclusive Healthcare Workforce

This cluster included statements regarding the demographics of healthcare providers as well as interpersonal qualities that would facilitate satisfying experiences for Black women seeking sexual healthcare. “More healthcare workers who are Black women” pointed out the demographic gaps in the healthcare workforce. Additionally, statements such as “healthcare providers being aware of Black women’s mistrust of the healthcare system” communicated a necessary orientation toward the unique historical relationship that Black women have with medical providers. Other statements focused on healthcare providers using active listening skills and believing the concerns of Black women in an effort to demonstrate themselves as trustworthy.

### Intimate Partner Roles and Responsibilities

The statements in this cluster focused on the relationships that Black cisgender women have with Black cisgender heterosexual men. Statements mentioned HIV testing, the availability and use of PrEP, and treatment adherence for men living with HIV. Two statements, “inclusive marketing” and “HIV prevention marketing by Black people for Black people,” specifically indicated a need to engage the Black community in HIV prevention messaging for the benefit of Black women. Additionally, “partner testing” and “mutual disclosure of HIV status among sexual partners” were statements that underscored the importance of relational approaches to HIV prevention. Additionally, the statements in this cluster spoke to the value of intentional, culturally responsive HIV efforts, such as considering the sexual health of Black men, “integrating HIV prevention and education into community organizations (e.g., churches),” and ensuring that “PrEP [is] offered in places used by Black women."

### Community Call to Action: Accountability

Building upon and expanding the previous cluster, the statements in the Community Call to Action: Accountability cluster emphasized the collective care of peer, family, and partner support for Black women engaging in sexual health and HIV prevention services. Being able to discuss PrEP options with a sexual or romantic partner and “peer support for Black women taking PrEP” indicated the interpersonal nature of the decision to initiate and continue PrEP. This cluster also addressed HIV and PrEP stigma: “less judgment from partners, friends, and family about taking medication.” This cluster emphasized the position that Black women occupy in relation to others in their community, providing a keen reminder that for this population, HIV prevention services must be specifically designed for women within a relational context.

### Prevention Resources and Education Programs

Access to information and resources as a key element of HIV prevention for Black women emerged in the Prevention Resources and Education Programs cluster. Corresponding statements advocated opportunities for dialogue about sex and sexuality, including “open sexual health discussions with minors” and “normalized conversations about sexual health and HIV.” Additionally, statements in this cluster included online sexual health resources, such as information about HIV testing and HIV risk assessment tools. This cluster also included statements addressing HIV testing services that are discrete and in proximity to spaces occupied by Black people.

### Public Health Interventions

Building on the previous cluster, the Public Health Interventions cluster outlines efforts that public health officials can take to address Black women’s sexual health and HIV prevention needs. These include clear communication regarding “the root causes behind health disparities involving Black women,” “ingredients and side effects of PrEP,” and the costs associated with PrEP. Other statements in this cluster include specific interventions such as home testing kits, needle exchange programs, and “sexual health resources for minors (e.g., access to condoms, birth control, dental dams, PrEP/PEP).” Related to the statement above regarding health disparities, “all PrEP options available to cisgender women” underscores the differential treatment of cisgender women in PrEP drug trials that has resulted in fewer choices for them in comparison to individuals assigned male at birth.

### Holistic Healing for Black Women

The final three clusters of the concept map directly address Black women, with Holistic Healing for Black Women encompassing multiple dimensions of wellness. “Ending the ‘strong Black woman’ stereotype” and “mental health counseling” are two statements that communicate the connection between psychological well-being and sexual health. “Financial stability for Black women” and “empowerment for Black women” speak to the value of both economic and personal autonomy, whereas “community for Black women” and “support for Black women at home” serve as reminders of the collectivism evident in Black communities. Additional statements in this cluster, focus on health directly and include health literacy, safe spaces to discuss sexuality, and “Black women using prevention methods when having sex with a man with HIV.”

### Black Women, Self-Care Is a Radical Act

Black women, Self-care is a Radical Act emerged as both a cluster and a mantra. Corresponding statements advocated the intentional prioritization and empowerment of Black women caring for themselves. This prioritization would look like black women “loving themselves,” “doing what makes them happy,” “being comfortable discussing sexuality,” and “taking time to rest.” Broadly this cluster focused on “peace for Black women” who must be “seen as fully human.”

### Personal Responsibility and Access to Healthcare

The final cluster addressed both personal and structural factors related to Black women’s sexual health. Corresponding statements included “routine STI testing” and “preventative wellness checks,” responsibilities of Black women that require them to be both proactive and consistent in order to maintain their sexual health. Yet, there is an acknowledgement within this cluster that Black women’s personal responsibility for their sexual health does not occur in a vacuum. Other statements included “access to care for Black women,” “more healthcare options for Black women,” and “HIV prevention methods that women can control.” These statements indicate structural and contextual factors that encourage or discourage Black women to seek sexual healthcare and that shape Black women’s ability to receive care appropriate to their needs.

### Pattern Match Graph

Once the cluster map was structured, a pattern match graph was generated to visualize the relationships among the ratings of the clusters [28] The stakeholder ratings used to generate the pattern match graph were high (i.e., above 3.5 on a 5-point scale) for importance, feasibility, and impact. The importance ratings ranged from 3.81 to 4.45, with Personal Responsibility/Access to Healthcare as the highest rated cluster in this domain. For feasibility, Prevention Resources and Education Programs rated the highest; however, overall, the clusters were rated lower in this area (range 3.49 to 4.05). In terms of impact, stakeholders rated Personal Responsibility/Access to Healthcare and Intimate Partner Roles/Responsibilities as the highest in this area with the range of all clusters being 3.61 to 4.39. The high and somewhat compact nature of the ratings required discussion with the stakeholders to determine priorities and a path to action.

## Discussion

Through a series of stakeholder meetings, we engaged community members in the GCM process to establish community-derived solutions to the problem of HIV’s disproportionate effect among Black cisgender women in Orange County, FL. The map that resulted from this process included nine clusters that indicate action steps that can be taken within the local community to facilitate satisfying healthcare experiences for Black women and enhance HIV prevention services. These steps will necessarily engage individuals and organizations from multiple sectors, including healthcare providers, HIV-serving organizations public health professionals, and community members who are invested in the health and well-being of Black women.

Two of the nine clusters (The Healthcare Provider’s Role and Qualities of an Inclusive Healthcare Workforce) included statements directly addressing healthcare settings and healthcare providers. Through these clusters, stakeholders emphasized the importance of environments and interpersonal experiences that disrupt the gendered racism that has historically marked Black women’s engagement with medicine [8]. Similar to previous research indicating that a friendly atmosphere and welcoming staff are facilitators of PrEP usage [25], these clusters identified ways that providers can work toward the creation of inclusive spaces for Black women. Moreover, these clusters aligned with previous studies that indicated the need for PrEP knowledgeable providers who acknowledge Black women’s medical mistrust and actively work to demonstrate themselves as trustworthy [17, 19]. PrEP knowledge among healthcare providers was also addressed through the cluster titled Public Health Interventions, which along with the Prevention Resources and Education cluster, indicated the need for improved training in medical programs, increased opportunity for continuing education, and community-based initiatives to facilitate accurate conversations about sexuality and sexual health. Prevention Resources and Education was rated the most feasible cluster despite legislative restrictions on sexual health content in Florida public schools [35]. Participants seemed to be advocating for community-based efforts to address gaps in sex education provided by schools to destigmatize and normalize discussions of sexuality, HIV, and PrEP.

The remaining clusters of the map were directed toward Black women and their social networks. The overarching message found in these clusters is that Black women need to prioritize their holistic well-being, but to do so, their partners, community, and the healthcare system must combine to establish the context where this is possible. For instance, Black women have reported that PrEP usage is a form of self-care given that they are often the one responsible for HIV prevention in their relationships [25]. Similar sentiments emerge in the clusters Personal Responsibility/Access to Healthcare and Black Women, Self-Care is a Radical Act; however, the clusters must be understood in connection to the Intimate Partner Roles and Responsibilities cluster which directly calls on Black men and other sexual partners to engage in HIV testing, disclose their status, and adhere to their prevention or treatment regimens. Black women are embedded members of their communities; hence, their efforts toward mental health, sexual wellness, and health literacy occur within the context of those communities. The clusters Community Call to Action: Accountability and Holistic Healing for Black women echo previous research findings regarding the key role of partners, friends, and family supports in supporting HIV prevention and PrEP usage among Black women [17, 18, 20].

### Next Steps: Healthcare Symposium

During the discussion of the concept map with our stakeholders, we broached the questions of what to do next and how to share the map with interested parties able to put the map into action. As researchers, we assured the stakeholders that the findings would be disseminated through the usual scholarly channels, such as peer-reviewed journal articles. However, we agreed with the stakeholders that the local healthcare providers and local community members needed a designated time and space to engage in candid, informative dialogue about Black women’s experiences in healthcare, sexual health needs, and HIV prevention concerns. The stakeholders proposed a symposium where providers and community members could come together to hear about the nine clusters of our concept map and to learn from each other about tangible ways to support Black women’s health. After that discussion, a group of stakeholders continued to work with us to plan a two-day symposium organized around these nine clusters which took place in February 2025.

### Limitations and Future Research

As with all research, there are limitations to this study that must be acknowledged. First, although the stakeholder group included a committed group of individuals from various backgrounds, the lack of gender and racial diversity within the group is a limitation. All stakeholders were women, and most were Black, which means that the process lacked input from men who might be sexual partners of Black women vulnerable to HIV. The perspectives of actual and potential partners could be valuable, especially given that the “Intimate Partner Roles and Responsibilities” cluster of the map directly referenced this group. Similarly, the process might have benefited from additional input from healthcare providers. Although some stakeholders reporting working in healthcare, most did not. A larger sample that included men and more healthcare providers might have provided more insight into their perspectives and roles in advancing sexual health equity for Black women. Like participants in previous studies [33], our participants rated most items high for importance, making prioritization of the clusters and the development of an action plan challenging. A larger sample might have provided more direction in this respect through ratings of impact and feasibility.

These limitations notwithstanding, the findings from this study offer a potential map to ending the HIV epidemic among Black cisgender women in a priority jurisdiction that has been understudied. The engagement with community stakeholders who have the lived experience relevant to the topic is an asset of this study and provides groundwork for future research. The concept map itself indicates potential next steps, including healthcare provider training related to Black women’s health and healthcare concerns, tailored PrEP programming for Black cisgender women in Orange County, FL, designated spaces for Black women to talk and learn about HIV and PrEP, and couples testing initiatives. Currently, few of these interventions exist in the region, and the findings from this study provide the foundational rationale for the initiation of such efforts.

## Conclusion

The sexual health and HIV prevention needs of Black cisgender women in Orange County, FL have gone unexplored despite them being a key population in efforts to end HIV in the region. Through group concept mapping we engaged with a group of community stakeholders who helped generate a map with nine clusters of action items for healthcare providers, community members, and Black women themselves. The findings of this study provide a roadmap for training, education, community action, public health initiatives, and holistic healthcare for Black women with the goal being a reduction in new HIV diagnoses within this population.

## Data Availability

The data that support the findings of this study are available from the corresponding author upon reasonable request and with sufficient explanation of the intended use of the requested data.
